# Advances in Environmentally Friendly Techniques and Circular Economy Approaches for Insect Infestation Management in Stored Rice Grains

**DOI:** 10.3390/foods12030511

**Published:** 2023-01-22

**Authors:** Inês Gonçalves de Sousa, Jorge Oliveira, António Mexia, Graça Barros, Carina Almeida, Carla Brazinha, Anna Vega, Carla Brites

**Affiliations:** 1 National Institute for Agricultural and Veterinary Research (INIAV), I.P., Av. da República, 2780-157 Oeiras, Portugal; 2 Linking Landscape, Environment, Agriculture and Food (LEAF) Research Center, Tapada da Ajuda, 1349-017 Lisboa, Portugal; 3 Ernesto Morgado S.A., Rua Prof. Casimiro de Oliveira 21, 3090-833 Barra, Portugal; 4 School of Engineering and Architecture, University College Cork, T12 YN60 Cork, Ireland; 5 Instituto Superior de Agronomia, Universidade de Lisboa, Tapada da Ajuda, 1349-017 Lisboa, Portugal; 6 LAQV/Requimte, Department of Chemistry, NOVA School of Science and Technology, FCT NOVA, Universidade NOVA de Lisboa, 2829-516 Caparica, Portugal; 7 Grain Technik Pvt. Ltd., B-2/12, Mohan Co-Operative Industrial Estate Badarpur, New Delhi 110044, India; 8 GREEN-IT Bioresources for Sustainability, ITQB NOVA, Av. da República, 2780-157 Oeiras, Portugal

**Keywords:** rice, non-chemical, treatments, prevention

## Abstract

Rice (*Oryza sativa* L.) is a staple food for about half of the world’s population. Therefore, it is important to search for solutions that minimise losses and production costs for producers and ensure food quality and safety for consumers. Improved methods for the detection and monitoring of hidden infestations are useful for adopting infestation control measures. Chemical methods are used to prevent rice losses due to infestations; changing this situation, however, is of the utmost importance, as it harms the environment and human health. The management of infestation by controlled storage conditions, namely temperature and atmosphere composition and the use of current fossil-based packaging with modified atmospheres, is well recognised. The use of environmentally friendly solutions is promising, but it is necessary to perform a life-cycle assessment and cost analysis to evaluate their effectiveness. According to the principles of circular economy, the integration of the best-selected treatments/solutions for insect management, along with the use of biopackaging from rice by-products are recommended. This review describes the methods of detection and control of infestation as well as several promising alternatives to chemical treatments; however, more research is needed in order to obtain effective technological solutions that can be applied at an industrial scale.

## 1. Introduction

Losses and wastage may occur at every stage in the paddy-to-rice process, from the farm to the consumers’ homes [1]. In addition, there is an increasing concern about improving rice production and minimising crop losses, as a response to the demands of the growing population worldwide. It is, hence, very important to understand the rice wastage origins and minimise rice losses that occur due to insect infestation.

Damages and losses in stored rice can be direct (quantitative/physical loss of grains) or indirect (qualitative/loss in quality and nutrition) [2]. Insect pests promote weight loss of the product stored, a very important factor, causing nutritional value loss, commercial loss, as well as quality degradation [3]. Insect damage can be significant when rice is stored for long periods, and insect populations reach high levels. Insect activity results in an increase in temperature and water content in the grain bulk and can promote deterioration of the grains [4].

However, these damages are difficult to estimate and depend on several factors, such as temperature, relative humidity, atmospheric conditions, and the storage duration, as well as the intrinsic properties of the various rice varieties. Therefore, in case of inappropriate storage conditions due to unscientific management, rice can be an ideal substrate for different types of contaminations of larvae and/or live insects.

Postharvest procedures have the main objective of maintaining the quality of the grain and preventing contamination by insects or fungi, and hence, the choice of that procedure can influence the rice quality [5]. If rice is stored in cool temperatures and dry conditions, eggs that might still exist will not hatch [4], hence the advice to store rice in cool and dry conditions. However, a rice miller has little to no control over storage conditions in clients’ warehouses and consumers’ homes.

Meanwhile, the use of pesticides on the field and fumigants in grain storage units to control infestations are still common, using mainly chemical agents (commonly fumigations) during storage [6]. Fumigants are being used worldwide, but their overuse is causing environmental problems, leading to a development of insect pests’ resistance [7], while chemical residues affect public health [8]. Fumigants generate residues of chemical contaminants that compromise the natural quality of rice and its products, and consumers are increasingly concerned about insecticide residues in food products [7].

Apart from the development of resistance in insects, there is a growing increase in restrictions with regards to the use of chemical fumigants, which are being replaced by alternative solutions in managing insect pests in stored food products to protect the food quality and the environment. Biopesticides appear as novel eco-friendly tools, and the adequate implementation of them could be a great alternative to protect stored grain against pests [9].

The search for solutions to prevent insect infestations and the evaluation of their effectiveness are important aspects for the sustainability of the rice value chain, impacting food products derived from rice and other cereals to a large extent.

The objective of this review was to report the common insects identified in rice grains, the methods of insect detection, and infestation-managing strategies. The potential alternative solutions to the traditional chemical fumigations were emphasised, identifying the most promising to mitigate rice losses due to insect infestation. In addition, developments with biopackaging from rice by-products were reported. In this way, the abundant rice by-products, which are traditionally waste, are reused and valorised, leading to waste reduction, in a circular economy approach.

## 2. Rice Insect Infestation

Insect infestation is a major cause of production loss, so the origin of pests, the intensity of their attacks, and the damage they can cause are considered important aspects for protecting stored products.

The origin of insects in stored grain has been debated for years, and although some insects have been found to infest the grain on the field, residual populations within bins are generally accepted as the primary cause of infestations [10]. Some life stages of insect pests can survive for very long periods of time [11], and the longevity of common insects identified in rice grains is relatively long comparing with other stored-grain pests [12]. It is thus of the utmost importance to properly clean all equipment related to the paddy to rice process, such as harvesters, trucks, conveyers, storage bins, as well as the milling equipment and the surroundings, to avoid contamination with insects, since the remaining old grain and dust build an ideal breeding ground for infestations. Yaseen et al. [13] suggested maintaining sanitation and ventilation at field and storage; cleaning and disinfection of the storage structure; gunny bags and bins followed by fumigation to avoid the carry-forward of infestations; as well as the filling of the cracks, crevices, and burrows as preventive measures for infestation. Champagne [10] concluded that proper cleaning and treatment of empty facilities should be the first and is perhaps the best treatment to prevent insect damage in stored rice.

Inside the grains, insect movement can be determined by seasonal conditions and grain temperature. This means that in the months when temperatures are higher, insect infestations will occur more on the surface of the grain, while when temperatures are low, infestations will occur more in the centre of the grain. In the later situation, insect infestations may not be detected early until the insects are present in large numbers [14].

All stages of the insect life cycle occur inside the grains. Some species of common insects identified in rice grains are the rice weevil (*Sitophilus oryzae* L.), granary weevil (*Sitophilus granarius* L.), lesser grain borer (*Rhyzopertha dominica* F.), and angoumois grain moth (*Sitotroga cerealella* O.), which are considered primary pests [14]. These insects that arise during rice storage can develop inside the grain (internal or hidden infestation) or outside the rice grain (external infestation), feeding on the bran, dust, or broken grains.

*Sitophilus oryae* and *Sitotroga cerealella* are the most relevant indoor infestations for stored rice and are part of a set of insect species that pierce the grains and reduce them to flour. *S. oryzae* and *Sitophilus granarius* are the most common and are quite similar, but each has unique physical characteristics and capabilities. Adult rice weevils have long snouts with chewing mouth parts at the end. A female that has been fecundated will chew a hole in the kernel with her long snout and excavate a small cavity into which she places an egg, subsequently sealing the hole with a gelatinous plug. An infected kernel is almost indistinguishable by the naked eye. Usually, only one egg per kernel will develop into larvae, but an adult female can lay about 400 eggs in her lifetime. The rice weevil usually lays more eggs than a granary weevil. Eggs hatch in a few days under favourable conditions but may stay dormant until such conditions are set (namely, temperature and humidity). The complete metamorphosis from egg to larvae, then pupa, and finally adult inside the kernel takes 35 to 40 days in favourable conditions, after which the adult chews its way out. The rice weevil is one of the most widespread and destructive insect pests found in stored cereals worldwide, and the interaction with rice involves all life stages of the insect, with the larvae being the most destructive stage. These insects cause rice losses and affect their quantity and quality [2].

*Rhyzopertha dominica* is yet another one of the most damaging insects that generally infest rice grains. Infestations caused by these insects are difficult to detect since larvae and pupae develop inside infested grains [15]. The chances of infestations of stored rice by this insect are increased as it can fly easily. Both the adults and larvae of *R. dominica* feed on rice grains, the adults externally and larvae internally, and in the case of large infestations, the grain can develop a musty odour and may also become heavily soiled with excrements.

*Sitotroga cerealella* is the most abundantly found grain moth in paddy rice storage. Usually, moth infestations affect the upper layers of stored grains in bulk, limiting the direct losses that this insect can cause. These insects can also infest the grains on the field, and most of the damage is provoked by larvae inside of the rice grains [15], since neonata larvae emerging from eggs begin feeding inside the grain where the life cycle developed.

Some specific conditions, such as adequate moisture content and temperature, increase the possibility of insect growth and, therefore, the occurrence of infestation [16]. Accordingly, it is important to control environmental parameters during storage, and in addition to researching solutions to prevent insect infestation, it is also necessary to determine the best detection methods for hidden infestation.

## 3. Detecting and Monitoring Tools

The search for solutions to detect and monitor the internal (hidden) infestation is of greater relevance for the rice processing industry because the grain is typically stored with husk (paddy rice), whose structure protects against external insects.

The most conventional techniques to inspect grain for internal infestation resort to the methods described in ISO 6339-4:1987 [17] describing a total of 5 methodologies for estimating the degree of, or detecting the presence of, hidden insect infestation. These include determining carbon dioxide production; the ninhydrin method; the whole-grain flotation method; the acoustic method; and the X-ray method. The principles of these methods will be explained below, and information will be provided on recent advances that can improve the performance of the different techniques.

### 3.1. Determination of Carbon Dioxide Production

The determination of carbon dioxide is based on the fact that the amount of carbon dioxide in storage is largely correlated to insect infestation (as a result of the insect respiration rate) [18], considering that the metabolic rate of dry grain is very low. It has been shown that mature larval instars of grain weevils produce more carbon dioxide than adults and that the accumulation of carbon dioxide in infested grain samples during 24 h is easily measurable by CO_2_ sensors [19]. However, it is also known that moisture content and temperature can interfere with carbon dioxide release in grain, especially because they can potentiate the growth of fungi, such as *Aspergillus* spp., which can produce significant amounts of carbon dioxide (exceeding the amounts produced by insects) [20,21]. Therefore, increases in carbon dioxide can often result from fungal spoilage instead of a hidden infestation. Advances in carbon dioxide determination have been made regarding wireless sensors that resort to machine learning algorithms for real-time monitoring and early warnings on possible grain infestation/spoilage [22].

As such, a modern sensor can help in the prediction and detection of incipient or ongoing spoilage/infestation with good accuracy.

### 3.2. Ninhydrin Method

The ninhydrin method, on the other hand, is based on a colourimetric reaction of ninhydrin, originally yellow, with a free α-amino group of primary amino acids, producing a purple-coloured dye known as Ruhemann’s purple. When an infested dry grain is crushed, the amino acids from the insect’s body fluid will react with ninhydrin in the paper surface, resulting in a purple spot. Amino acids of the grain are not released and do not react. The number of spots indicates the level of hidden infestation [17]. Not many developments have been reported on this method, and this technique is less amenable to automation compared with the determination of carbon dioxide.

### 3.3. Flotation Method

The whole-grain flotation method relies on the fact that internal insect infestation reduces grain mass, making the grains float. When sound and infested grains are immersed, the sound ones will sink, while the infested ones will float to the surface. The flotation method has found a good implementation worldwide, and adaptations of this method have been reported to detect insect fragments in bran, fine bran, and flour [23,24]. However, this method is time-consuming, and results are of qualitative but not quantitative value, mainly because the method is most likely to produce an underestimate of the level of infestation present in the sample.

### 3.4. Acoustic Methods

Acoustic methods for insect infestation detection rely on identifying the sound patterns of the targeted insects. An acoustic vibration sensor, connected to an amplification system, will transmit the noise caused by the feeding activity of hidden insects. The use of acoustic technology in insect pest management applications has increased significantly between 1980 and 2010 [25]. Different acoustic devices are currently commercialised for detecting hidden insect infestations [25,26]. Advances in this system include digital signal processing and statistical analysis tools, such as those based on neural networks or machine learning/deep learning, to distinguish targeted pests from each other and from background noise [27,28]. These advances enable the automated monitoring of the abundance and distribution of pest insects in stored grains, which might greatly impact the value of future commercial solutions.

### 3.5. X-ray Imaging Method

X-ray imaging techniques are based on the exposure of a one-grain thickness layer of rice to soft X-ray, followed by inspection to identify insects within the grains. The use of X-rays has many advantages since it is a fast, non-destructive, and accurate technique for the internal and external detection of insects, regardless of the life stage of the insect. Furthermore, recent algorithms focused on X-ray image contrast enhancement, or microcomputed tomography for 3D imaging, enable superior diagnostic images and, consequently, high accuracy [29,30]. Nevertheless, the automatic inspection of insect infestation is still a challenge. In this regard, deep learning methods, in particular, artificial neural networks and convolutional neural networks, have been applied to differentiate between infested and non-infested maize grains; this knowledge is likely to be applicable to rice infestation as well [31,32].

In addition to the most conventional techniques, other approaches are focusing on optimising the testing time, improving the accuracy, and/or applying non-destructive techniques. Near-infrared spectroscopy (NIRS), able to detect the presence of specific volatile compounds produced by insects, chromatography, or mass spectrometry techniques (performed after solid phase extraction), or more recently, the use of electronic noses that sense specific volatile compounds, are good examples of recent developments that have been used in this research field [26].

### 3.6. Polymerase Chain Reaction Technique

Molecular-based techniques, detecting specific genetic regions of a target species, are another good example of recent developments for infestation identification. These techniques have emerged in the last decades due to their accuracy, detection limit, specificity, and high throughput capabilities. Among the diverse technologies, the polymerase chain reactions (PCR), in particular, those based on quantitative approaches (qPCR), have gained great relevance. However, although the PCR is routinely used in the food industry to detect foodborne pathogens or genetically modified organisms (GMOs) in foods, its use to detect insect presence is still at an early stage. With regards to grains/cereal infestation, a few research works use a PCR and insects’ species-specific DNA regions to accurately detect and even quantify early infestation [33]. Examples can be found for internal/hidden and external infestation and different grains/samples.

A study by Nowaczyk et al. [34] on developing a real-time PCR method for detecting *Tribolium confusum* infestations in stored products has shown the method’s ability to detect infestations as low as 1 insect per kg of oat flakes. Further, the detection of external infestation with *Tribolium castenaum* by a quantitative (qPCR) method has been validated in wheat flour, showing a detection limit of 0.046 adult insects in 5 g of wheat flour [33]. These works clearly show the potential of this technique to quantify infestation to very low levels. Considering the FDA defined the maximum permissible limit of insect fragments in flour of 75 or approx. 3 adults per 50 g of flour [33], the value of PCR techniques becomes quite obvious. These techniques could be easily implemented as routine techniques, as happened in auto control systems in food safety or in adulteration testing. The qPCR’s capabilities/facilities are currently part of the technique’s repertoire of food control laboratories, and the qPCR usually presents a time-to-results of 24 h or 48 h, depending on which timeframe can fit into the dynamics of the companies. Similar to food safety control plans, sampling schemes could be put in place to take representative samples from large lots and take into consideration the risk assessment of each company. Nonetheless, those techniques require a laboratory setup, so they do not replace in situ sensors; however, they can certainly add relevant, highly accurate, and complementary information.

In 2016, a real-time PCR method was developed to identify hidden infestations of *R. dominica* in grain (rice, maize, and wheat) [35]. Later on, the study was extended for the five most relevant internal insects pests, making the work of Sòla et al. [36] paradigmatic of the PCR potential for detecting hidden infestations. A multiplex PCR was developed and tested in different grains, including rice. Insect species included *R. dominica*, *S. granarius*, *S. oryzae*, *S. zeamais*, and *Sitotroga cerealella*. The estimated detection limit was 0.1 pupa per kilogram of rice, except for *R. dominica* (10 pupae per kilogram).

As such, taking into consideration the specificity and limit of detection of these detection techniques, as well as the broad distribution/use of PCR instruments in food-related laboratories, it is expected that the industry can resort more often to these techniques. However, their potential for application in situ for real-time monitoring is limited, as they require laboratory settings. Hence, methodologies such as those based on acoustic or carbon dioxide sensors, which are more amiable in in situ-automated monitoring, are expected to find a higher degree of dissemination in industrial settings.

## 4. Management Strategies

### 4.1. Fumigation Methods: Limitations and Health Concerns

Fumigation is a chemical treatment and one of the most effective methods for controlling insects’ growth in stored cereal grains [7]. This method can be used against several species of insects and is inexpensive [37], quick, and easy to apply [16]. If properly applied, the treated paddy or rice would remain in a container or silo for the sufficient time to kill the living insects, but fumigation will not destroy the eggs. Fumigation hence has to be repeated in certain intervals. Alternatively, the temperature of the grain has to be lowered to a level where insects become dormant, or at least to a level where their life cycles are significantly prolonged and hence their reproduction is extremely slow. Rice kernels damaged by the infestation will be removed in the sorting process.

Despite the success of fumigation, resistances in insect pests have been identified [38]. Furthermore, fumigation is not always carried out properly, for example, an Australian Grains Research and Development Corporation (GRDC) survey in 2017 revealed that only 49 per cent of users applied phosphine correctly in Australia. In addition, due to concerns regarding health risks and ozone layer depletion, some of the most potent fumigants have been banned already [16], and it is important to find alternative treatments. Fumigants applied to control pest populations can have adverse effects and be toxic to humans [37], and some stored-product pests have developed resistance to them [38].

Fumigation with phosphine or methyl bromide was the most used pesticide in stored-product pests [16]. However, with all the problems associated with the use of phosphine (chemical residues, resistant insect species) [7,37,38] and the regulations related to methyl bromide which was phased out [39], these products are being used less. There is an increasing preoccupation with replacing chemical treatments. According to the European Commission, since 2009, the number of low-risk or non-chemical pesticides approved for pest control has doubled [40].

A deltamethrin-incorporated polypropylene bag (ZeroFly^®^ Storage Bag) has been developed (Vestergaard SA, Lausanne, Switzerland), claiming great potential to reduce postharvest losses in cereal grains and grain legumes [41]. Although the study found that the bags were efficient in preventing insects from entering the bags, it seemed to leave those already inside unaffected, so their usefulness is doubtful. k-obiol is another pesticide produced by Bayer^®^ (Leverkusen, Germany), which has recently been used in the industry as an alternative to phosphine. Unpublished trials, though, were unconvincing regarding its performance for fumigation compared to phosphine. Therefore, its impregnation in packages would likely give unsatisfying results as well.

However, the continued use of these pesticides increases the risk of exposure [6]. There has been an increasing concern worldwide in substituting chemical treatments with methods of biological origin, since chemical pesticides have a tremendous impact on biodiversity, the environment, as well as animal and human health. It is thus important to identify viable solutions that could minimise the use of insecticides and reduce their impact on the environment [42].

### 4.2. Control by Environmental Parameters

Critical environmental parameters such as temperature and atmosphere (extrinsic factors) affect the storability of rice because they can cause problems related to insect infestation and other biological changes and contaminations. Therefore, product temperature and storage atmosphere control are critical to prevent rice losses related to those problems and to estimate the most significant risk periods [43].

#### 4.2.1. Temperature

Grain temperature is one of the most important factors in controlling insect infestation and monitoring the temperature of rice grains is mandatory for maintaining quality throughout storage [44].

Temperature control prevents insect infestation in rice storage [10], since insects cannot survive or thrive outside a temperature range of 13–35 °C [45]. Although treatments using high temperatures can control pests in stored products, they can lead to quality degradation [37]. Bringing the grain temperature to a level where a heat treatment using hot air is effective is very difficult since the grain would dry, and the evaporative cooling would keep the rice temperature much lower than the applied air temperature. Hence, heat treatment with hot air would lead to an undesirable, substantial over-drying of the grain. Furthermore, any isochoric heating of the grain that would positively affect insect infestation would significantly change the product properties and lead to discolouration triggered by the so-called Maillard reaction [46], as observed, and in these cases, would be desirable during paddy steaming and parboiling.

On the other hand, cooling rice, with or without refrigeration, has been shown to be effective against insects [10]. There are many storage situations where ambient conditions are insufficient to cool the grain; hence, refrigerated air units for chilling grain have been developed for these situations [47]. During the last 60 years, chilled aeration (around 2–5 °C) has been successfully used and applied commercially to preserve grain quality [48]. Maintaining low temperature and moisture levels in bulk-stored grain was identified in a major study on “Enhancing the quality of U.S. grain for international trade” [49] as the main way to preserve grain quality and to prevent damage from moulds and insects as early as 1989.

##### Grain Chilling

In actuality, grain chilling is the most used technology in the rice industry to remove excess heat after harvest or drying, significantly preserving the quality of the rice stored and allowing long-term storage regardless of the ambient conditions.

In grain chilling, grain is cooled using a mobile refrigeration system that controls the temperature and relative humidity of the aeration air independent of the ambient conditions [48].

Although storage temperatures lower than 5 °C have been recommended in the literature [50], it has been shown that keeping the grain temperature below 20 °C reduces the development rate of insects compared to 25 °C product temperature [51].

Several studies could prove that using grain chilling in industrial silo complexes can keep the stored paddy insect-free, even for extended periods and extreme weather conditions, if the product temperature is kept at 15 °C or below. Therefore, a storage temperature of below 15 °C is recommended, and 20 °C grain temperature should not be exceeded. Lazzari et al. [52] found that chilling a 5.000 t metallic paddy silo to 15 °C in Brazil controlled the insect populations for about 60 days without re-chilling. Similarly, Lazzari et al. [53] reported that after initial chilling to 12–14 °C, stored rice in a huge rice facility in Brazil kept its temperature for about 60 days without re-chilling. When the rice was kept at this temperature level it was found free of external insects after 8 months of storage.

Furthermore, these studies highlight that once the grain has been initially cooled, “only occasional re-chilling for short periods is required to maintain chilled storage conditions due to the insulating properties of the grain itself” [48].

A simulation carried out for a paddy silo in Costa Rica [51] revealed that it would take less than 5 days to cool the product to a top layer temperature of 14.6°C, and that once cooled, the average grain temperature would only increase to 15.5°C over a storage period of 6 months, despite the high local ambient temperatures. Since the average grain temperature remained within the range where insect development would stop [45], the need for chemical control of the stored-product insects would reduce or be eliminated.

Chilling grain below 15 °C in less than a week avoids that most insect species complete one life cycle because they take at least a month to develop from egg to adult at ideal temperatures (30–35 °C) [54]. Morales Quiros [51] concluded that “chilled aeration is the only technically feasible strategy to achieve average grain temperatures sufficiently low to reduce or eliminate the need for chemicals to control stored product insects”.

Since only occasional re-chilling is required after the initial chilling, grain chilling can be an economical solution for chemical-free pest control, even in tropical conditions. Morales Quiros [51] found the operational cost of grain chilling to be lower than the combined cost of aeration with ambient air and fumigation. Even in moderate climates in Europe, the use of grain chillers can be cheaper than the use of aeration fans when the weather is unfavourable for longer periods (for example, during the unusually wet summer of 2021 in southern Germany), given the extremely long time to bring down the grain temperature using aeration fans in this case.

It is also important to note that chilling not only hinders the growth of pest populations, but also allows the avoidance of quality losses in cereal storage and product deterioration [4].

Lazzari et al. [53], however, highlighted the importance of proper cleaning of the storage facility before storage for successful chemical-free and insect-free paddy storage using a grain chiller. A one-time phosphine fumigation cycle followed by grain chilling to 15 °C is widely used in the industry if living insects are present before storage, and it has proved effective for insect-free long-term storage. It is important to remember that phosphine fumigation is ineffective at low temperatures and must be carried out before the grain is cooled.

Although the cost of grain chilling is higher than the cost of aeration with ambient air [55,56], it is lower than the cost of ambient aeration and fumigation combined [57,58], making it an economically feasible method for chemical-free pest control. It can be concluded that grain chilling, which preserves the quality and quantity of the product stored, is an efficient method for insect control, even during long-term storage and independent of the ambient temperature fluctuations.

#### 4.2.2. Storage Atmosphere

The control of the atmospheric composition to protect grain products, such as rice, has been extensively reported [59,60,61,62,63]. Insect eggs would not hatch under certain conditions, such as the absence of oxygen. Therefore, whether in a silo or a small consumer package, if the environment is hermetic and has no oxygen, an infestation will not occur.

There are several options for controlling the rice grain’s surrounding atmosphere:

**Vacuum packaging.** A low-pressure environment where all air is removed and the packaging material ensures hermeticity will also protect from humid storage environments (high water vapour barrier); therefore, an infestation will not occur at the consumer stage. Although vacuum packaging is more expensive than the usual packaging since the packaging material must be more resistant and requires a vacuum packaging machine, it is an efficient option to prevent the growth of insects.

**Hermetic packaging.** This option requires a more expensive packaging material than usual, with a very high barrier to gas permeance, and is cheaper than the option above as it would not require vacuum packing. This technology avoids interactions with the surrounding environment, can extend the shelf life during storage, and maintains the food quality [61]. If eggs were present, their development would consume oxygen, and the growth would stop. This is known as passive modified atmosphere packaging (the modified atmosphere is created by the metabolisms ongoing in the product itself). This has been tested, for instance, by Guenha et al. [61], concluding that using hermetic packaging is safe, pesticide-free, and sustainable. It also contributed to a decrease in insect infestation. A particular type of bag (PICS—Purdue Improved Crop Storage bags), consisting of two inner layers of high-density polyethene and an outer layer of woven polypropylene, was reported to give excellent results by Martin et al. [62]. In this case, the results proved that wheat grains stored in the PICS bags had lower insect damage levels than in conventional packaging. Additionally, Baoua et al. [63] used PICS bags for stored rice infested with Tribolium spp. and *R. dominica*. In rice infested with *R. dominica*, the results showed 96% mortality after 2.5 months, and the number of insects did not increase above the initial value. Covele et al. [60] also studied hermetic containers as an alternative to preserving rice grains, since this method proved efficient for 12 months without using pesticides. The results showed that this could be a green alternative for safe rice storage with several advantages. Hermetic cocoons are another type of hermetic packaging that consist in two plastic halves joined together with an airtight seal, after being loaded with bags of stored rice. The control of insect grain pests without chemicals is the main benefit of this packaging, but if not managed correctly, rapid re-infestation by insects can occur [64].

**Active MAP.** Active modified atmosphere packaging removes the normal air and injects a different gas composition instead. It also obviously requires hermetic packaging, so the cost of this solution is higher than even vacuum packaging, as one has to add the cost of the gases. However, there are some cost-effective solutions to generate a modified atmosphere to inject into the packages. Several options have been reported in this regard (Table 1):

- **Carbon dioxide.** Carvalho et al. [69] found that the use of CO_2_-enriched atmospheres (90–95%) in storage silos and big bags successfully eliminated insect infestation. CO_2_ further has anti-fungicidal properties, contributing to solving an equally important problem faced by the rice milling industry. The CO_2_ treatment suppressed insects in eggs, early larvae, and adults and can be applied either in the final product, during the packaging process, or in other stages to preserve the quality and flavour of rice during storage [69]. Atmospheres containing about 60% CO_2_ rapidly kill stored-product insects, with about 4 days of exposure at 26°C, being sufficient to kill all stages (including eggs) of most stored-product insects [70].
- **Nitrogen.** The total removal of oxygen while maintaining nitrogen instead of the vacuum has been proposed [66,71,72] to implement in silos by using pressure-swing adsorption to gradually replace normal air with an environment rich in nitrogen (above 98%), extracting oxygen from the kernels themselves. The exposure times needed are longer than the ones that are currently in use for different fumigants, and the application of nitrogen in silos is a very complicated procedure, given that leaky structures should be thoroughly improved in their gas-tightness level in order for nitrogen to be successfully applied [73]. This makes the use of nitrogen for pest control in silos several times more expensive than the use of chemical fumigants; this is the reason why the use of nitrogen for fumigation is still mostly restricted to the storage of organic produce.
- **Ozone.** Ozone gas can be used for disinfestation and decontamination since it does not produce residues [37] and has important advantages compared to other methods, as this gas does not leave residues in food and is GRAS (Generally Recognised As Safe) [74]. Ozone would be used as fumigation in silos, which is unsuitable for packaging because ozone decomposes quickly, so it is necessary to keep on generating to maintain its concentration. Its use is described by Amoah and Mahroof [67]. The results reported by these authors are not very encouraging. While ozone can affect all stages of the insect life cycle, it very much depends on how deep in the kernel the egg is located, as the ozone effect is rather limited to the surface and close to it. Even with treatment for 60 h with high ozone concentrations, at depths of 15 cm and higher, there was still significant survival. Rice kernels are much smaller than this, so the treatment could be quite effective if applied in a fluidised bed for all eggs to be destroyed as they eventually hatch. Ozone also has some disadvantages as a stored-product fumigant as it is a strong oxidiser, and the effect of ozone exposure on silo materials needs to be assessed. It may increase corrosion rates on metal components and degrade equipment such as rubber seals and electrical equipment at unacceptable rates. It is highly doubtful that the use of ozone as a fumigant in grain storage could ever be used in an industrial scale, since ozone is highly climate active and other, proven fumigants, have already been banned due to this reason. Not directly related to contamination by insects but with the residues of chemical treatments, Ávila et al. [68] studied ozone gas as a degradation agent of pesticide residues in stored rice grains. The samples of rice treated with insecticides were exposed to the gas, and after ozonation, the quality of rice grains was not affected, and the technique was promising to remove insecticide residues in rice grains.

**Silica** has given good results in preventing insect development in cereals [75,76]. Initially, the use of cheap inert dust, such as volcanic ash, which is high in silica content (over 50%) has been proposed. However, there would be issues with the residues left from the dust that would now become part of the rice, which include the significant potential for off-flavours and would lead to insoluble particles floating as the rice was being cooked. Thus, Kar et al. [76] proposed a nanotechnology approach using silica nanoparticles. The treatment was considered effective, but there was a residual presence for all treatment conditions reported.

### 4.3. Essential Oils

Essential oils containing volatile compounds from plants are examples of chemical substitutes that can control and prevent rice losses due to insect infestations.

Essential oils could be an excellent alternative treatment to prevent biological contaminations in rice. Some essential oils extracted from a series of plants have shown significant antifungal and repellent properties, as well as insecticidal activity against stored-product pests. Natural products that would be organoleptically acceptable could be mixed with the rice and offer insecticidal action, but few options have been reported in this regard (Table 2). Garlic, for example, has well-known properties against insect infestation; however, its use would release strong flavours to the rice. Essential oils obtained from ginger, black pepper, or fennel could be used instead, providing less of an organoleptic impact. Their effectiveness was reported by Chang et al. [77], who used these different types of oil extracts in sachets instead of mixing them with the rice in order to avoid flavour impacts. While the sensory assessment proved no organoleptic impact, the fumigation efficacy was just around 80% at best. Basil oil has proven effective in killing rice weevils in open air and is thus suggested as a potential means to control the infestation. Follett et al. [78], however, reported a low impact on weevil mortality and reproduction rate when applied in packed rice. Essential oils obtained from basil, cinnamon, eucalyptus, mandarin, oregano, peppermint, tea tree, and thyme plants were studied by Hossain et al. [79], all individually, as well as combined. The results were positive, since all the essential oils showed toxicity against the rice weevil, with eucalyptus essential oil having the highest toxicity, causing 100% mortality at the minimum concentration. These authors also verified that combining oregano and thyme essential oils was more efficient than the individual treatments. Zargari et al. [80] proved that eucalyptus essential oils have insecticidal and repellent properties in insect control in stored grains. The compounds were characterised by gas chromatography-mass spectrometry (GC-MS), and their effectiveness has been evaluated by molecular docking and conventional molecular dynamic (CMD) simulation. The authors concluded that *Eucalyptus camaldulensis* essential oils are rich in insecticidal terpenes and can control *S. oryzae*.

Other studies about essential oils extracted from plants confirmed activity against insect’s metabolism. Guettal et al. [81] concluded that the essential oil derived from *Citrus limonum* exhibited fumigant toxicity against *S. granarius* adults, confirming its potential as a natural alternative to synthetic insecticides for the control of stored-product pests. To study the fumigant toxicity of *C. limonum* essential oil, after washing, the leaves were dried in the shade and ground into powder. The obtained oil then was dried over anhydrous sodium sulphate and was analysed via GC-MS. The components were identified based on the retention index compared with the reference mass spectra.

Orange oil has also been used as an alternative agent for controlling many insect pests due to its neurotoxicity to insects, as described in a study by Chou et al. [8]. The oil showed low mammalian toxicity and short environmental persistence. In addition, Mishra et al. [82] concluded that the essential oils of *Syzygium aromaticum* and *Aegle marmelos* could be recommended as an alternative to synthetic insecticides since they are inexpensive, easily available at the farm level, as well as environmentally sound with low mammalian toxicity. Finally, Bhavya et al. [83] showed that the essential oil of *O. tenuiflorum* had a significant fumigant activity against *S. oryzae,* concluding that this essential oil could be used in the formulation of biofumigants as a safer alternative to chemical fumigants.

Shi et al. [84] claimed very high efficiencies in preventing rice weevil infestation by using an emulsion of cinnamon oil with anhydrous ethanol, which prevented its otherwise rapid oxidation and loss of toxicity. However, there was no analysis of the potential organoleptic impact. Al-Harbi et al. [85] evaluated the insecticidal activity of *Ocimum basilicum*, *Nigella sativa*, and *Lavandula angustifolia* essential oils against *S. oryzae* by assessing the mortality percentage assay in the adult stage of the insect, as well as analysing genes associated with the toxicity effect of the essential oils. In 2014, Nenaah [86] tested the bioactivity of essential oils obtained from 3 different plants (*Achillea biebersteinii*, *A. fragrantissima*, and *Ageratum conyzoides*) as grain protectants and their insecticidal activity against *S. oryzae*, *R. dominica*, and *Tribolium castaneum*. This author obtained the composition of the oil using GC and GC-MS, and the results were positive since the plant species showed considerable toxicity against the tested rice pests.

Other authors [87] studied the efficacy of *Carlina acaulis* essential oil against several insects that attacked stored products, concluding that this essential oil has elevated pesticidal properties.

### 4.4. Biopesticides in Packaging

Biopesticides are frequently part of the natural defence mechanism of many plant species, usually showing high selectivity against target pests with low toxicity, as well as being biodegradable. They can be applied to protect crops and seeds, which can be seriously damaged by insect infestation during storage and transport, causing economic losses [88]. The use of biopesticides is increasing since regulation agencies set lower residual limits for synthetic pesticides and encouraged synthetic alternatives [89]. In addition, consumers are encouraging the replacement of chemical substances with biopesticides.

Biopesticides can be impregnated in the packaging material to create an anti-insect effect and avoid significant changes in the rice grain composition. This may work in killing adult insects, but it must be noted that the dead insects will remain inside the package. The insecticide is released from the package over a given time. This is the critical issue for the application to rice weevils, as an egg can take over a month to become an adult, which is then killable. Some options have been suggested in the literature:

**Terpenes.** Goñi et al. [88] impregnated low-density polyethylene films with supercritical CO_2_ and terpenes ketones and obtained a good result of 100% mortality in adult insects but only for up to two days, with the toxicity decreasing to very low levels in just seven days. The researchers developed these films to obtain a packaging material protecting seeds, kernels, and derivatives during storage and transport. Although this study was related to maize and its pests, it had a positive effect, and it would be desirable to verify its effectiveness on stored rice.

**Biopesticides in double-layered bags.** Soujanya et al. [90] proposed placing a biopesticide in between 2 layers of plastic for a double-layered bag with the biopesticide not being in contact with the rice. The biopesticide used in this study was the leaf powder of *Tinospora cordifolia*. The results showed good efficacy, supporting the concept of a broadened biopesticides approach as a control method.

**Chitosan.** Silva et al. [91] reported some fungicidal effects of chitosan-coated paperboard. However, the best efficiency in preventing insect infestation was under 80%.

The development of active packaging incorporating biopesticides is an innovative technology for food preservation, considering their antifungal, insecticidal, repellent, and herbicidal activities. Herrera et al. [9] obtained a bioactive material for stored-grain protection by incorporating 1-octen-3-ol in low-density polyethylene films (LDPE). The supercritical CO_2_-assisted impregnation of LDPE films with the biopesticide was carried out in a high-pressure cell, with magnetic stirring and a high-pressure impregnation system. The films developed by these authors indicated that this biopesticide had insecticidal activity against *S. zeamais*, showing 100% mortality after 24 h. Although *S. zeamais* is a maize pest, it is also common in stored rice, and it would be important to test the efficiency of the 1-octen-3-ol on rice samples contaminated with *S. oryzae*.

### 4.5. Application of Radiations

Radiations such as ultraviolet light, visible light, microwaves, infrared light, and radiofrequency waves can be used for disinfestation. Some studies on applying these radiations have already been carried out, obtaining very positive results. Duangkhamchan et al. [92] studied the use of infrared heating, consisting of an electrical emitter with adjustable intensity to tune the temperature against *S. oryzae* in an egg stage. The results showed 100% insect mortality after two minutes of exposure at all tested temperatures. Pei et al. [93] studied the lethal effects of infrared radiation on *S. zeamais* and *T. castaneum* in rice, concluding that heating the rice to 60 °C under infrared radiation of 2780 W/m^2^ could be a feasible method for disinfestation. The rice and insect samples were treated using a ceramic infrared drying device consisting of an infrared radiation emitter, a circulating fan, and a control panel. The rice and insects were heated using different infrared emitter temperatures. Then, the radiation intensity of the heated rice was measured, with the authors concluding that with this treatment, it was possible to achieve high insect mortality.

Other researchers studied the application of radiation as an alternative to conventional treatments. Follett et al. [94] used an irradiation quarantine treatment for stored-product pests, and the authors found that this treatment could control rice weevils. They randomly selected 15 insect adults and placed 500 g of rice in each of 20 plastic containers, which were treated with different radiation doses, counting the number of live and dead adults every week for five months. The conclusion was that a 120 Gy radiation dose could be used for this pest control method, and no further damage would occur to the rice grains.

Srivastava and Mishra [95] studied the application of microwave, ultraviolet light, and vacuum, as well as the combination of these three radiations in controlling the adult stage of *R. dominica* in rice grains. The analysis was conducted using microwave, ultraviolet irradiation, and temperature control equipment. The process and status of the reactions in a container were observed in real-time using an instant camera system. Their conclusions were that combining the three treatments yielded better results and led to minimal changes in rice quality attributes. Some years earlier, Zhao et al. [96] had used a microwave oven to study the effect on insect adults and eggs of rice weevils. The insects suffered 100% mortality at a temperature above 55 °C, with an applied microwave energy above 0.017 kWh/kg.

Radiofrequency electromagnetic waves have been proposed for stored-grain insect pest disinfestation by several authors. Radiofrequency technology is well-developed for various applications such as pasteurisation and rapid heating (similar to microwaves, just in different bandwidths). This method is a common non-chemical disinfestation process with effective and rapid action [37]. Organic materials such as insects contain high moisture and dielectric loss factors, and heat can be transferred rapidly under an electromagnetic field. When the energy is absorbed, the heat is generated rapidly in insects [97] and radiofrequency technology can be applied to eliminate all stages of the insect life cycle, from egg to adult. Vearasilp et al. [97] reported the construction of a simple radiofrequency heating pilot system wherein the rice fell through the radiofrequency field, reached temperatures of not more than 55 °C, and came out completely clear of contamination of all forms of the weevil life cycle, after just 1–3 min of treatment. The quality of the rice before and after cooking was determined by instrumental methods and showed no significant difference from that of untreated rice. This system is environmentally friendly, safe for consumers, and can eliminate the rice weevils at any stage, while no organoleptic assessment was verified. The application of this technology could be useful when applied to rice before it is conveyed into the storage silos and would potentially leave all rice free of infestation for the upcoming storage.

## 5. Biopackaging Derived from Rice By-Products

Rice by-products (rice bran, rice husk/hull, rice straw) are produced in abundance during the rice process. Traditionally, rice brain is mostly used for feed application, and rice husk is discharged or burned [98]. Therefore, there is a need to convert rice by-products from waste to added-value food biopackaging, with biodegradable properties. The resulting biopackaging protects food against light, humidity, and other contaminants and could contribute to an increase in revenues for the rice industry.

### 5.1. Rice Bran

Rice bran is the layer covering the white rice endosperm, which is removed during the milling process of brown rice. Rice bran is rich in many bioactive compounds, appealing to the following food applications: phenolic and cinnamic acids, anthocyanins, flavonoids, steroidal compounds such as tocopherols, arabinoxylan, as well as proteins [99]. Rice bran is commonly used as animal feed or for bran oil extraction.

As far as bioplastics are concerned, rice bran has a relatively high content of valuable protein (about 10–15% [100,101]) and starch. Starch is a suitable and common biopolymer for packaging, and its tensile properties are adequate for this application, with 50% of the commercial biopackaging being produced from starch [102]. Rice bran-based biopackaging typically comprises starch and protein, and a plasticiser, commonly glycerol or sorbitol [103,104]. Rice bran-based biopackaging has appealing thermoplastic properties and is produced by injection moulding, similar to current fossil-based packaging. Although rice bran oil is increasingly important in cosmetic, food, and pharmaceutical applications, it should be removed during the biopackaging formulation, as it contributes negatively to the mechanical properties of bioplastics [105].

### 5.2. Rice Husk/Hull

Rice husk covers the brown rice and is produced during the milling of paddy. Rice husk is composed mostly of very hard materials, such as lignin, hemicellulose, and cellulose, with known barrier properties to O_2_, and hydrated silica [99]. It is commonly incinerated to produce energy for several processes such as dryers with its ash being a low-cost product having a high silica content (83–90%). Rice husk ash is used to manufacture silica gels, silicon chips, activated carbon and silica, lightweight construction materials, zeolites, and lithium batteries [99].

Rice husk-based bioplastics are composed mainly of cellulose, and silica is used as the filler with cellulose [106]. Starch-based bioplastics with silica as the filler were demonstrated to be promising when compared to the currently used plastics [107].

### 5.3. Rice Straw

Rice straw is produced during harvesting as well as the threshing of the panicles and has a high content of cellulose. A bioplastic was produced based solely on the cellulose extracted from rice straw [108]. A composite bioplastic with starch as the matrix and the previously isolated cellulose nanocrystal CNC were formulated by casting with different starch-to-CNC ratios. Incorporating cellulose nanocrystals in the bioplastic increased the tensile strength and modulus but decreased its thermal stability [109]. Another composite bioplastic was proposed with cellulose from rice straw cellulose and chitosan and glycerol as additives. The increase in glycerol content led to a reduction in oil swelling and made a more flexible (higher elongation at break), and weaker (low tensile strength) bioplastic [110].

The reported bioplastics have promising properties, with already known biopolymers materials; however, to the best of our knowledge, the production of such biopolymers at the commercial-industrial scale was not yet reported.

## 6. Conclusions

The proper cleaning and treatment of empty facilities should be the first and is perhaps the best treatment to prevent insect damage in stored rice, since the remains of old grains build an ideal breeding ground for infestations. However, insect infestation during storage is nearly unavoidable, especially in favourable ambient conditions and for extended storage periods. The use of conventional methods for infestation control requiring chemical fumigants is becoming more and more stringent due to the potential risks to human health, tightening legal regulations, as well as increasing resistances in insects. Grain chilling is an established technology to reduce the temperature of stored paddy and rice, minimising any kind of storage-related losses. Although it is not feasible to bring the stored good to a temperature where insects would be killed, storing the grain below 20 °C significantly reduces the development rate of insects, and thus minimises grain losses due to insects as well as the fumigation requirement. The use of a controlled atmosphere (CO_2_ as well as nitrogen) to control stored-grain insects has been proven to be a feasible alternative to chemical fumigation. It is, however, several times more expensive than chemical fumigation, takes longer to achieve mortality in insects, and is difficult to achieve, since it requires air tightness of the storage bin. Other alternative treatments such as biopesticides, the use of ozone gas, radiofrequency, microwaves, ultraviolet light, and vacuum, as well as infrared heating have been explored as well. Some of these techniques could be viable options for environmentally friendly insect management, and a few methods furthermore show potential for the removal of insecticide residue in stored rice. The applicability of these technologies in the rice processing industry should be determined as a priority. Following the principles of the circular economy, in the future, biopackaging from rice by-products should be used along with appropriate treatments for insect management. Furthermore, a life-cycle assessment and cost analysis will be necessary to propose alternative methods and biopackaging for insect infestation management at a large scale.

## Figures and Tables

**Table 1:** Gaseous options of active modified atmosphere packaging used to manage insect infestation in stored rice.

Active MAP	Results	Reference
Carbon dioxide	CO_2_-enriched atmospheres successfully eliminate insect infestation. CO_2_ has antifungal properties and suppresses insect eggs, early larvae, and adults and preserves the quality and flavour of rice during storage.	Carvalho et al. (2019) [59]
Nitrogen	Complete (100%) mortality of *T. confusum* (all life stages), *O. surinamensis* (larvae and adults), *S. granarius* (L.) (adults), and *R. dominica* (adults).	Navarro et al. (2012) [65]
	Successful solution for the control of stored-product insects that are resistant to phosphine.	Sakka et al. (2020) [66]
Ozone	Affects all stages of the insect’s life cycle, but it depends on how deep in the kernel the egg is located.	Amoah and Mahroof (2019) [67]
	Efficient in removing insecticide residues in rice grains.	Ávila et al. (2017) [68]

**Table 2:** Studies related to the application of essential oils from plants to manage insect infestations in stored rice.

Essential Oil Origin	Results	Reference
Ginger, black pepper, and fennel	Sensory assessment proved no organoleptic impact. Fumigation efficacy around 80% at best.	Chang et al. (2017) [77]
Basil	Low impact on weevil mortality. Reproduction rate when applied in packed rice.	Follett et al. (2014) [78]
Basil, cinnamon, eucalyptus, mandarin, oregano, peppermint, tea tree, and thyme plants	Toxicity against the rice weevil with eucalyptus essential oil having the highest toxicity, causing 100% mortality (minimum concentration).	Hossain et al. (2019) [79]
*Eucalyptus camaldulensis*	Rich in insecticidal terpenes that can control *S. oryzae*.	Zargari et al. (2022) [80]
*Citrus limonum*	Fumigant toxicity against *S. granarius* adults.	Guettal et al. (2021) [81]
Orange oil	Low mammalian toxicity and short environmental persistence.	Chou et al. (2022) [8]
*Syzygium aromaticum* and *Aegle marmelos*	Inexpensive, and easily available at the farm level. Environmentally sound with low mammalian toxicity.	Mishra et al. (2013) [82]
*Ocimum tenuiflorum*	Fumigant activity against *S. oryzae*.	Bhavya et al. (2018) [83]
*Ocimum basilicum*, *Nigella sativa*, and *Lavandula angustifolia*	Cinnamon oil exhibited 100% repellent effect on rice weevil.	Shi et al. (2022) [84]
Basil, black seeds, and lavender	Lavender essential oil had the highest toxicity activity for rice weevils with 100% mortality effect.	Al-Harbi et al. (2021) [85]
*Achillea biebersteinii*, *Achillea fragantissima*, and *Ageratum conyzoides*	The essential oils from the 3 plant species exhibited toxicity against the pests of stored grains.	Nenaah (2014) [86]
*Carlina acaulis*	High pesticidal properties.	Kavallieratos et al. (2022) [87]

## Data Availability

Not applicable.
